# One Pot Synthesis, Biological Efficacy of AuNPs and Au-Amoxicillin Conjugates Functionalized with Crude Flavonoids Extract of *Micromeria biflora*

**DOI:** 10.3390/molecules28083320

**Published:** 2023-04-09

**Authors:** Kamran Jalil, Shabir Ahmad, Nazrul Islam, Rahim Ullah, Qudsia Jalil, Sulaiman Sulaiman, Anoosha Sajjad, Riaz Ullah, Ali S. Alqahtani, Ahmed Bari, Hidayat Hussain, Essam A. Ali

**Affiliations:** 1 Chemistry Department, Islamia College University, Peshawar 25000, Pakistan; 2 Department of Chemistry, Government Degree College Hayatabad, Peshawar 25000, Pakistan; 3 Department of Pharmacy, Sarhad University of Science and Information Technology, Peshawar 25000, Pakistan; 4 Department of Pharmacy, University of Peshawar, Peshawar 25000, Pakistan; 5 Department of Pharmacognosy, College of Pharmacy, King Saud University, Riyadh 11451, Saudi Arabia; 6 Department of Pharmaceutical Chemistry, College of Pharmacy, King Saud University, Riyadh 11451, Saudi Arabia; 7 Department of Bioorganic Chemistry, Leibniz Institute of Plant Biochemistry, Weinberg 3, D-06120 Halle (Saale), Germany

**Keywords:** crude flavonoids, *Micromeria biflora*, Au−amoxi conjugate, anti-inflammatory, antinociceptive

## Abstract

Amoxicillin is the most widely used antibiotic in human medicine for treating bacterial infections. However, in the present research, *Micromeria biflora’s flavonoids* extract mediated gold nanoparticles (AuNPs) were conjugated with amoxicillin (Au-amoxi) to study their efficacy against the inflammation and pain caused by bacterial infections. The formation of AuNPs and Au-amoxi conjugates were confirmed by UV–visible surface plasmon peaks at 535 nm and 545 nm, respectively. The scanning electron microscopy (SEM), zeta potential (ZP), and X-ray diffraction (XRD) studies reveal that the size of AuNPs and Au-amoxi are found to be 42 nm and 45 nm, respectively. Fourier-transform infrared spectroscopy (FT-IR) absorption bands at 3200 cm^−1^, 1000 cm^−1^, 1500 cm^−1^, and 1650 cm^−1^ reveal the possible involvement of different moieties for the formation of AuNPs and Au-amoxi. The pH studies show that AuNPs and Au-amoxi conjugates are stable at lower pH. The carrageenan-induced paw edema test, writhing test, and hot plate test were used to conduct in vivo anti-inflammatory and antinociceptive studies, respectively. According to in vivo anti-inflammatory activity, Au-amoxi compounds have higher efficiency (70%) after 3 h at a dose of 10 mg/kg body weight as compared to standard diclofenac (60%) at 20 mg/kg, amoxicillin (30%) at 100 mg/kg, and *flavonoids* extract (35%) at 100 mg/kg. Similarly, for antinociceptive activities, writhing test results show that Au-amoxi conjugates produced the same number of writhes (15) but at a lower dose (10 mg/kg) compared to standard diclofenac (20 mg/kg). The hot plate test results demonstrate that the Au-amoxi has a better latency time of 25 s at 10 mg/kg dose when compared to standard Tramadol of 22 s at 30 mg/ kg, amoxicillin of 14 s at 100 mg/kg, and extract of 14 s at 100 mg/kg after placing the mice on the hot plate for 30, 60, and 90 min with a significance of (*p* ≤ 0.001). These findings show that the conjugation of AuNPs with amoxicillin to form Au-amoxi can boost its anti-inflammatory and antinociceptive potential caused by bacterial infections.

## 1. Introduction

One of the promising fields in the health sector is the use of nanomedicine as an alternate solution to tackle antibiotic resistance [1]. Green nanoparticles are gaining much of the attention [2] due to their lower toxicity [3] and intrinsic physicochemical properties [4,5], which helps them to be used in antimicrobial resistance [6], drug delivery [7], *DNA* analysis [8], gene therapy [9], and in biosensors [10]. The synthesis of environmentally friendly Ag and AuNPs has been reported using *Onion* [11], *Pelargonium graveolens leaves* [12], *Azadirachta indica* leaf [13], and *clove* extract [14]. Similarly, Ling, L et al. explored the anti-Parkinson’s disease properties of AuNPs using *Cinnamomum verum* extract [15]. Nanoparticles and their drug conjugates have received attention due to their ability to enhance drug efficacy and minimize its side effects at much lower doses [16]. AuNPs have gained much of the attention due to their conjugation with antibiotics to increase their efficacy against bacteria [17]. In a recent study, it has been reported that vancomycin-conjugated gold nanoparticles have shown more efficacy than vancomycin itself against resistant bacterial strains [18]. Similar studies reveal that colistin-conjugated AgNPs have shown more efficiency than AgNP*s* and colistin against Gram-positive bacteria [19]. The synthesis of metal nanoparticles using plant extracts or compounds extracted from plants has received much attention in the last two decades [20]. Cao-Milan et al. investigated the potential biomedical applications of gold nanoparticles based on their chemical inertness, surface properties, photosensitivity, and electronic structure [21].

Sandri et al. used FeNPs to enhance the efficacy of ibuprofen (IBU) via conjugation [22]. The anti-inflammatory potency of gold and silver nanoparticles (NPs) was explored by Priyanka Sing et al. using an extract of *Prunus serrulat* fruit [23]. Fahad A. Al-humaydhi et al. reported *Saffron*-stigma-extract-mediated AuNPs and studied their anti-inflammatory and analgesic potential [24]. CeONPs have shown a decrease in lung infection and fibrosis in the Bleomycin Murine Model when conjugated with micro-RNA46a [25]. Wang Zhao et al. reported Trimethylated chitosan polyglycolide nanoparticles as a drug carrier for delivery to the brain [26]. Nano formulation of cefixime using AuNPs has been reported to combat the resistance strain of *Escherichia coli* and *Klebsiella pneumoniae* [27].

Similarly, Qi et al. has reported that the use of nanoparticle conjugates to treat ovarian cancer is safe [28]. Over the years, nanoparticles have been used to deliver drugs to lung tissue and cancer cells [29]. Yahyaei, Behrooz and Pourali, Parastoo recently published results of biologically inspired AuNPs conjugated to anticancer drugs in one step without the use of additional linkers [30]. Similarly, Wang et al. used *Coccinia grandis* bark extract-prepared AuNPs, conjugated to the drug N-acetylcamosine (NAC), and evaluated its anticancer properties [31]. Recently reported, both Ag and AuNPs chemically synthesized by the green route exhibit excellent antibacterial activities [32]. Sheikh et al. have reviewed the mechanistic insight of nanoparticles action against multidrug resistance bacteria [33]. Amoxicillin is one of the oldest and most often suggested antibiotics. It is a bactericidal and works well against most Gram-positive bacteria. It has been reported that the use of amoxicillin can reduce the inflammation and histological changes induced by bacterial infections [34]. However, in recent years, it has developed resistance to some common bacterial strains. It has shown resistance to helicobacter pylori, a major cause of gastric cancer in developed countries [35].

In the present study of biogenic AuNPs, Au-amoxicillin conjugates were synthesized and then their effects against inflammation and pain caused by common bacterial infections were evaluated using in vivo anti-inflammatory and antinociceptive models. The NPs and its amoxi conjugates were characterized using Uv–vis spectroscopy, Fourier-transform spectroscopy (FT-IR), energy dispersive X-ray (EDX), X-ray diffraction (XRD), scanning electron microscopy (SEM), thermogravimetric analysis (TGA), and zeta potential (ZP).

## 2. Results

### 2.1. Synthesis of AuNPs and Au-Amoxicillin Conjugate

Different volumes (12, 14, 16, 18, and 20 mL) of 1 mM gold solution were taken in to 1 mL of *Micromeria biflora* flavonoid extract, and the mixture was stirred at room temperature for 1 h. Formation of AuNPs was demonstrated by a color change (yellow to ruby red) upon addition of 1 mL of flavonoid extract. Color change is also supported by UV–vis absorbance at 535 nm (characteristic region of AuNPs). Optimal ratio (18:1) was selected for further study. Au-amoxi formation was confirm by plasmon bands at 545 nm.



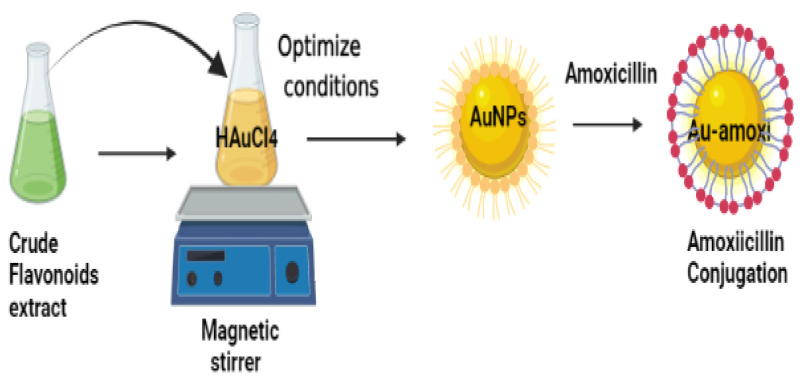

A schematic representation of AuNP and Au-amoxi synthesis.

#### UV–Visible Spectroscopy (Uv–Vis)

**Figure 1:**
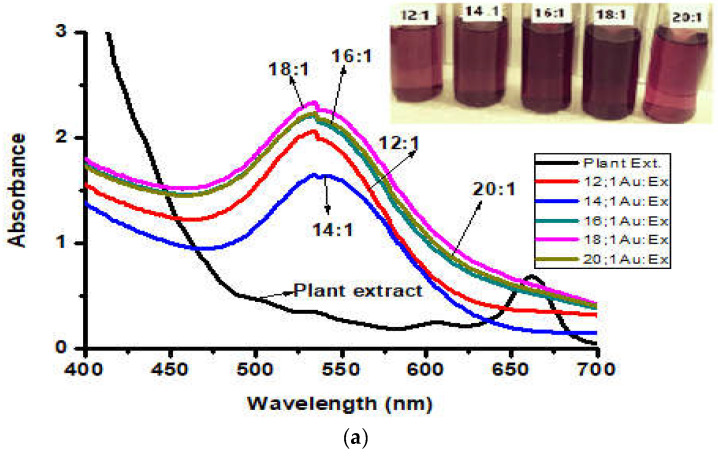

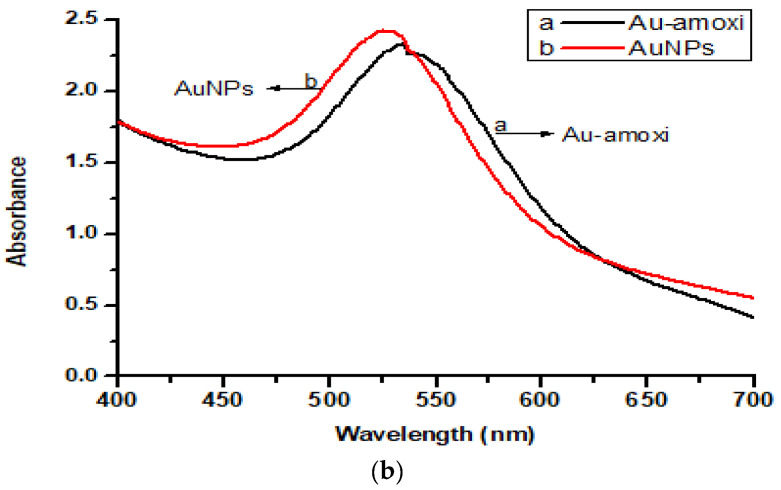
(**a**) Uv–visible spectra of AuNPs synthesized using flavonoid extract of *Micromeria biflora*. (**b**) Uv–visible spectra of Au-amoxi conjugate.

The formation of AuNPs and Au-amoxi was monitored through UV–visible spectrophotometer at a wavelength between 200–800 nm. (Figure 1a) shows different absorptions between 535–550 nm with variable intensities indicating different shape and size of the AuNPs. The formation of the gold amoxicillin conjugate (Au-amoxi) demonstrated by Uv–vis results showed absorption at 545 nm at a longer wavelength compared to AuNPs (Figure 1b). This may be due to the exchange of plant moieties with drug molecules, during which aggregation may occur, shifting the absorption to a longer wavelength.

### 2.2. Fourier-Transform Infrared Spectroscopy (FT-IR)

**Figure 2:**
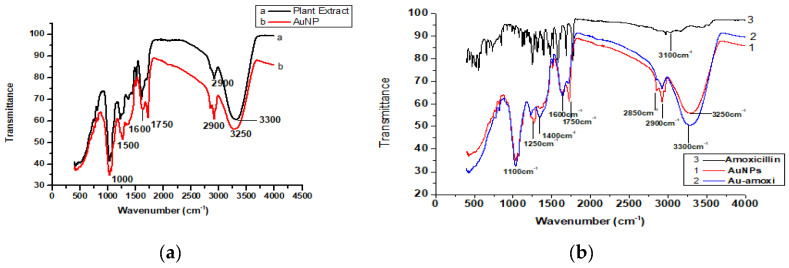
(**a**) FT–IR spectra of flavonoids extract of *Micromeria biflora* and AuNPs; (**b**) FT–IR spectra of AuNPs and Au–amoxi conjugate.

FT-IR studies were performed to identify possible entities involved in the reduction, stabilization, and capping of AuNPs. A comparison of FT-IR spectra of prepared *Micromeria biflora* flavonoid extract and AuNPs was performed. In (Figure 2a) (curve a), plant material shows a high intensity band at 3200 cm^−1^, indicating (–OH stretching) of alcohol, phenol, or carboxylic acid of flavonoids responsible for the reduction of gold ions. A band of low intensity at 2850 cm^−1^ shows (–CH_2_) groups. Similarly, a band at 1600 cm^−1^ for C=O stretching. A weak band at 1200 cm^−1^ indicates the C-O stretching of esters, cyclic ethers, alcohols, or carboxylic acids. In (curve b), (Figure 2a) shows the possible involvement of all these functional groups during formation, stabilization, and capping of AuNPs by shifting all these band slightly. Similarly, the FT-IR spectra (Figure 2b) of AuNPs and Au-amoxi conjugate (curve 2) show slight shifting of IR bands, indicating the conjugation has taken place and amoxicillin has linked to the surface of AuNPs through van der Waal interactions of NH_2_ S, OH, or carboxylic acid groups present in the amoxicillin. A wide band at 3100 cm^−1^ in amoxicillin spectrum (curve 3) shows the presence of hydrogen-bonded OH or NH_2_ group, while during conjugation, this band is more sharper and appears at 3300 cm^−1^. A lot of changes have been observed from 400 cm^−1^ to 2000 cm^−1^ in amoxicillin spectra when conjugated with AuNPs. The comparison between IR spectra of AuNPs (curve 1) and Au-amoxi (curve 2) reveals the absorption of OH or NH_2_ group is slightly shifted from 3250 cm^−1^ to 3300 cm^−1^, while peaks at 1750 cm^−1^ shift to 1600 cm^−1^, which is equally supported by the presence of a peak at 1250 cm^−1^, which may be due to in-plane bending of OH group or due to the presence of C-N stretching. The band at 1750 cm^−1^ in AuNPs indicates the presence of a carbonyl stretching which is equally supported by a band near 1150 cm^−1^ of C-O starching.

### 2.3. Scanning Electron Microscopy (SEM)

**Figure 3:**
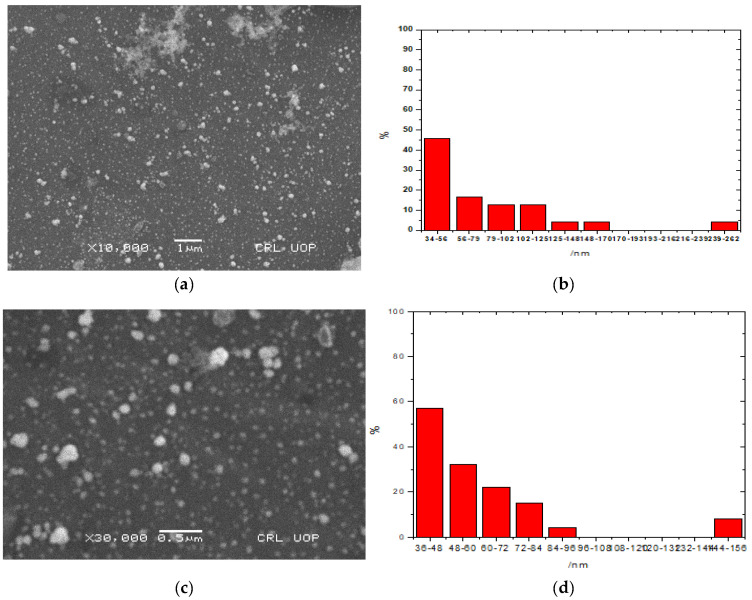
(**a**) SEM image of AuNPs functionalized with crude flavonoids extract of *Micromeria biflora*; (**b**) size distribution percentage of AuNPs functionalized with flavonoids extract of *Micromeria biflora;* (**c**) SEM image of Au–amoxi; (**d**) size distribution percentage of Au–-amoxi.

SEM analysis shows that most of the particles (AuNPs and Au-amoxi) are spherical (Figure 3a–d). The average particle size is between 42–78 nm (AuNPs) and 45–113 nm for Au-amoxi conjugates. This is in good agreement with the UV–visible results. Particle size distribution of AuNPs (Figure 3b) shows that the particle sizes range from 34 nm to 262 nm, while 47% of the particle size ranges from 34–56 nm. Similarly, the Au-amoxi size ranges from 36 nm to 156 nm (Figure 3d), and 57% of the particle size ranges from 36–60 nm.

### 2.4. XRD, EDX, and TGA Analyses

**Figure 4:**
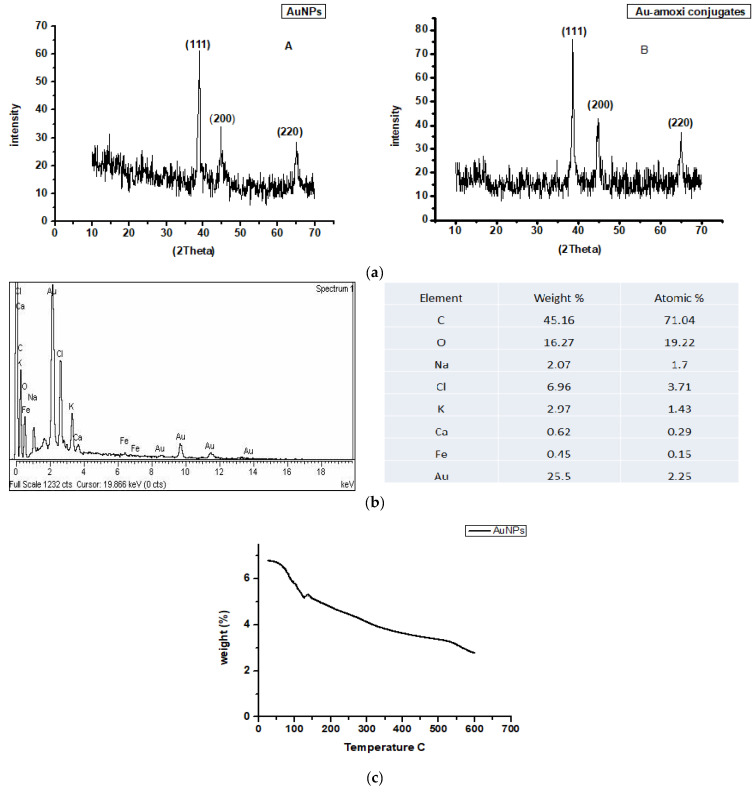
(**a**) XRD analysis of AuNPs (A) and Au-amoxi conjugate (B). (**b**) Elemental analysis (EDX) of AuNPs functionalized *Micromeria biflora*. (**c**) TGA analysis of AuNPs functionalized *Micromeria biflora*.

XRD analysis is a method that is use to find the size and crystalline nature of the samples. XRD of AuNPs (Figure 4a) showed three distinctive diffraction peaks in the 2θ range of 10°–70° at 38.2°, 44.3°, and 64.6°, respectively, which indexed the planes (111), (200), and (220) of the cubic-face-centered gold. The cubic-face-centered structures of gold matched those in the database of the Joint Committee on Powder Diffraction Standards, USA (JCPDS no. 96-900-8464) [36]. The size of the AuNPs and Au-amoxi conjugate was calculated using “Scherrer” equation which shows that the size of AuNPs is around 35 nm, while that of Au-amoxi conjugate is around 49 nm, which is quite consistent with other characterization techniques. Similarly, elemental analysis (EDX) of AuNPs shows the formation of NPs and its conjugate (Figure 4b).

The thermal behavior of the AuNPs sample was studied by TGA at temperatures from 0−650 °C. (Figure 4c) shows that in the beginning at 24–129 °C, 1.73 mg or 25% weight loss occurs. While, at 30–512 °C, most of the organic matter lost and 1.92 mg, or 36.64%, weight reduction was observed. This indicates that the number of phytochemicals that covers AuNP ranges from flavonoids to small ones and may help in the synthesis and stabilization of AuNP having been removed. Finally, when the compound was furnace to 513–650 °C, a further weight loss of 0.56 mg, or 16%, was recorded that shows AuNPs are highly stable.

### 2.5. Zeta Potential

**Figure 5:**
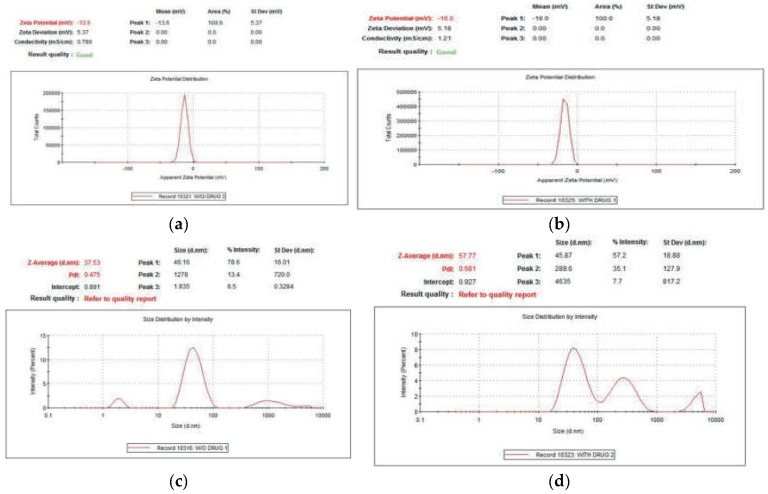
(**a**) Zeta potential of AuNPs functionalized with crude flavonoids extract of *M. biflora*; (**b**) Zeta potential of Au-amoxi conjugate. (**c**) Size distribution of AuNPs. (**d**) Size distribution of Au-amoxi conjugate.

Colloidal solutions with zeta potential (ZP) values ranging between ±30 mV are highly stable [37]. The present study results reveal that the ZP of AuNPs is −13.6 mV with a standard deviation of 5.37 mV and that of Au-amoxi is −16.0 mV with standard deviation of 5.18 and are fairly stable according to the reported literature [38]. The ZP value was found to fall in the negative side, i.e., −16 for Au–amoxi, which showed the effectiveness of the amoxicillin molecules as a capping agent by providing an extensive negative charge and thus preventing agglomeration [39]. The particle size distribution of AuNPs with a z- average of 37.57 nm and pdi of 0.475 suggests the polydispersity of the sample. Similarly, the z-average of Au–amoxi is 57.77 nm with a pdi of 0.681, indicating that Au–amoxi has more size distribution compare to AuNPs. The results indicate that the average size of Au–amoxi is more than AuNPs; this may be due to the replacement of some of the phytochemicals with amoxicillin molecules resulting in an increase in size of Au–-amoxi. This is equally supported by the UV–vis and SEM results (Figure 5a–d).

### 2.6. Effect of pH and NaCl on AuNPs Stability

**Figure 6:**
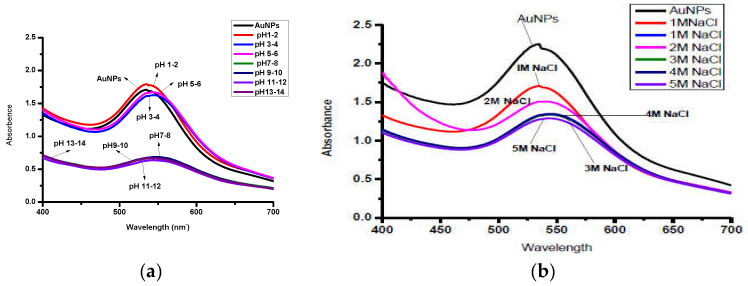
(**a**) Effect of different pH on AuNPs functionalized with crude flavonoids extract of *Mi*–*cromeria biflora*; (**b**) effect of various concentrations (1–5 M) on stability of AuNPs.

The pH was adjusted to 2–14 using 1 M HCl and NaOH, respectively. AuNPs are found to be stable at acidic pH (5–6) and less stable under strongly basic conditions (Figure 6a). Aggregation of NPs begins when the pH changes from acidic to basic. The effect of different concentrations of NaCl 1–5 M (Figure 6b) shows that the absorption of AuNPs shifts towards longer wavelengths with increasing concentrations of NaCl, sug–gesting the change in shape and size of NPs with changing electrolyte concentrations.

### 2.7. Biological Efficacy

#### 2.7.1. Inflammatory and Antinociceptive Activities

**Figure 7:**
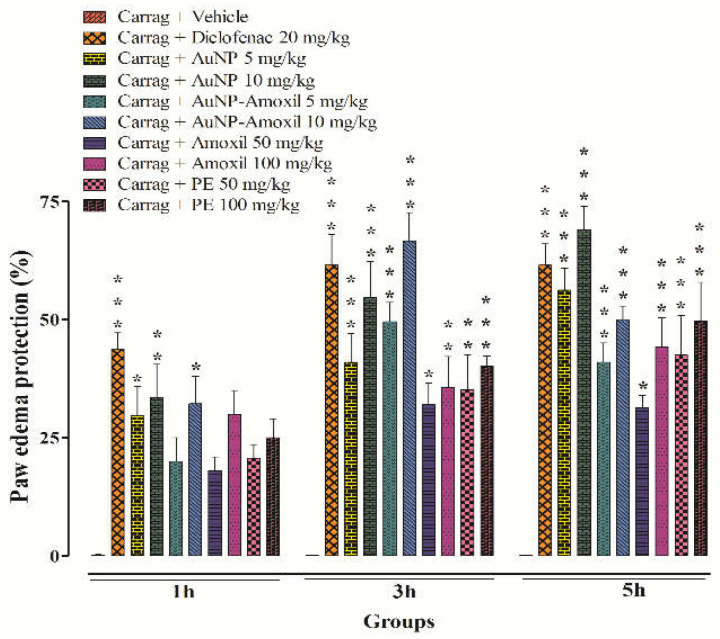
Effect of AuNPs, AuNP-Amoxi conjugate, CFE, and Amoxicillin on carrageenan-induced paw edema in rats. Bars represent mean ± SEM. * *p* < 0.05, ** *p* < 0.01, *** *p* < 0.001 compared to the vehicle treated animals. Data were analyzed by one-way ANOVA followed by Tukey’s test.

The in vivo inflammatory results reveal that AuNPs-amoxi at 10 mg/kg exhibited substantial anti-inflammatory activity (*p* < 0.001), compared to the vehicle treated group. The maximum percent inhibition of test compounds AuNPs (53%) and Au-amoxi (70%) was shown at a dose of 10 mg/kg body weight as compared to standard diclofenac (60%) at 20 mg/kg, while amoxicillin and P.E showed (30% and 35%) at higher doses (100 mg/kg). These findings show that the test compounds can be used to stop the second stage of inflammation [40] (Figure 7).

#### 2.7.2. Writhing Test

**Figure 8:**
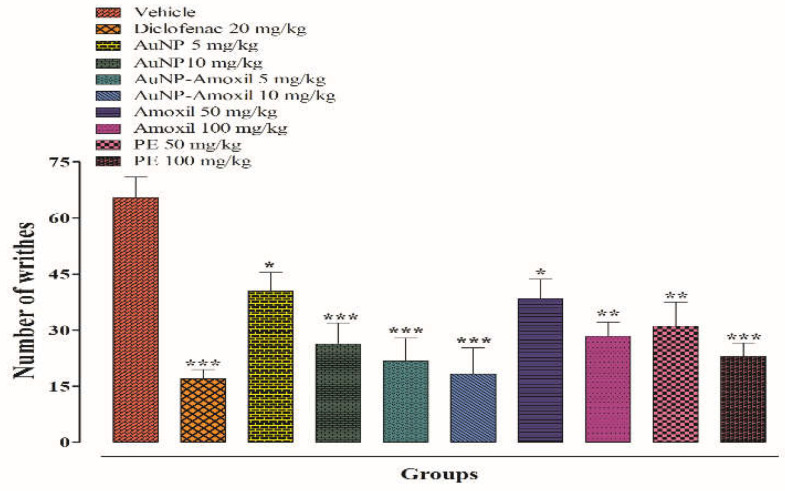
Chemically induced antinociceptive pain (writhing test). Bars represent mean ± SEM. * *p* < 0.05, ** *p* < 0.01, *** *p* < 0.001 compared to the vehicle treated animals. Data were analyzed by one-way ANOVA followed by Tukey’s test.

A dose-dependent approach was followed for entire compounds. The number of writhing produced was counted after 20 min, and it was noted that the Au-amoxi conjugate decreased the number of writhings with significant antinociceptive activity (*** *p* < 0.001). The results show that Au-amoxi conjugates produce 15 writhes at a dose of 10 mg/kg as compared to standard at a 50 mg/kg (Figure 8). These results show that Au-amoxi conjugates can be used as pain-relieving drugs in the future. 

#### 2.7.3. Hot Plate Test

**Figure 9:**
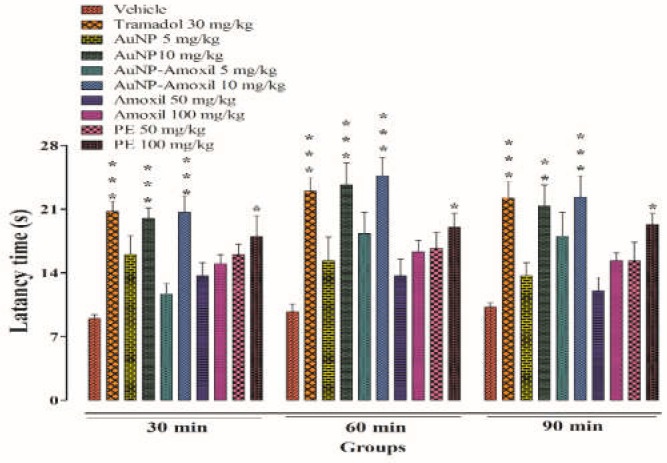
Thermally induced antinociceptive pain in mice using hot plate. Bars represent mean ± SEM. * *p* < 0.05, ** *p* < 0.01, *** *p* < 0.001 compared to the vehicle treated animals. Data were analyzed by one-way ANOVA followed by Tukey’s test.

The hot plate test, also known as the thermal analgesia trial, is a sort of trial that may be performed to determine the antinociceptive property of trial substances. The findings of the experiments show that a dose of 10 mg/kg of Au-amoxi and AuNPs had a longer latency time after 30 min than the vehicle, Tramadol, amoxicillin, and P.E at 30 mg/kg, 50 mg/kg, and 100 mg/kg, respectively. When animals were kept on a hot plate for 1 h, it was noted that latency duration was substantially longer for Au-amoxi than for other test compounds, and it decreased marginally after 90 min. The latency period for the mice to lick their rear paws or jump out of the plate is longer for Au-amoxi conjugate, which might be attributed to the nanoparticles’ capacity to release the drug at a faster rate (Figure 9).

## 3. Discussion

The therapeutic remedies of the plants have been talk of the town from ancient time. In recent years, green nanoparticles have emerged as an alternative to conventional medicine in the fight against pathogenic diseases and as a drug carrier [24]. Plant-mediated metallic nanoparticles gained importance not only because of their applications but also due to their eco-friendliness, cost effectiveness, and easy handling [41]. In the present study, AuNPs were synthesized using flavonoids extract of *Micromeria biflora* as a reducing and capping agent. Studies have revealed that shape, size, and dielectric constant of the medium are responsible for optical properties of nanoparticles which are one of the most interesting areas for researchers [42]. The formation of AuNPs has been confirmed by color change due to the collective oscillation of electrons (SPR) on gold surfaces [43]. The plasmon peak for AuNPs appears to be at 535 nm and for Au-amoxi conjugates at 545 nm. This may be due to the fact that during the conjugation some of the phytochemicals/flavonoids detached and were replaced with amoxicillin molecules, resulting in an agglomeration of particles and, hence, the absorption at a longer wavelength (Figure 1a,b).

The FT-IR studies show that polyphenols, which are the chief constituents of flavonoids, are accountable for gold reduction. In the absence of additional strong ligating agents in adequate concentration, flavonoids adsorb on the surface of metal nanoparticles, which might be due to contact through carbonyl groups. It is also conceivable that polyphenols contribute to metal ion reduction by oxidizing aldehydic groups in molecules to carboxylic acids [15]. The total quantity of phytochemicals capped the AuNPs is determined by TGA analysis [44]. The analysis demonstrates that the overall quantity of phytochemical-capped AuNPs spans from flavonoids to tiny protein molecules, which may have aided in the creation and stability of AuNP*s*. The weight changes for the synthesized AuNPs are shown in the third step at a temperature range of 513–600 °C [45]. The SEM results reveal that the size of Au-amoxi conjugates is more compared to AuNPs and, besides spherical shape, some nano rods or nano triangles may be present. The XRD results reveal that both AuNPs and Au-amoxi conjugates have crystalline structures with a cubic shape and are in good agreement with the reported data [46]. The EDX analysis is used to calculate the elemental ratio of a mixture and shows a high percentage of gold by weight. The zeta potential results provide an extensive insight for the stability of both AuNPs and Au-amoxi. The ZP value for AuNPs of −13.6 mV shows that flavonoids as a capping agent provide enough negative charge to make them sufficient, stable, and prevent aggregation [47]. The stability of gold nanoparticles at acidic pH shows that some of the moieties oxidized and become more effective capping agents; thus, making the colloidal solution more stable. 

Antibiotics are usually used to overcome the pain and inflammation caused by bacteria; however, over the years, these drugs are mostly associated not only with gastrointestinal problems [48], but also generated some resistance to common pathogens; thus, alternative medicines have received a lot of attention. AuNPs and their amoxicillin conjugates (Au-amoxi) have a highly significant impact in this investigation at both phases of inflammation, especially at a dosage of 10 mg/kg. The inflammatory activities carried out using the carrageenan paw edema model revealed that the inflammatory responses occurred in two stages. Histamine and serotonin are produced in the initial phase of inflammation, followed by the production of prostaglandins and bradykinin in the latter phase of inflammation [49]. AuNPs and Au-amoxi conjugates were found effective in both phases. The findings imply that flavonoids extract of *Micromeria biflora* might be useful in the treatment of pain and inflammation, as flavonoids are responsible for the suppression of COX−2 enzymes in addition to their antioxidant effects [50]. The acetic acid writhing test indirectly releases the endogenous mediators that encourage the neurons sensitive to most drugs [51]. Prostaglandins sensitize nociceptors, which causes writhing to occur [52] by the action of *cyclooxygenase*-1(COX-1) and its isoform which produced hyperalgesia ending pain. In this work, gold nanoparticles and Au-amoxi conjugates significantly reduced acetic acid-induced abdominal contraction when compared to diclofenac sodium at all dosages examined. This test is useful for the evaluation of mild analgesic non-steroidal anti-inflammatory compounds. The hot plate test shows more latency time for the Au-amoxi conjugate which may be due to the fact that the amoxicillin-incorporated nanoparticles work more efficiently and can release drugs faster.

## 4. Experimental Section

### 4.1. Materials and Methods

All chemicals and reagents used in this experiment were of analytical grade. Merck hydrogen tetrachloroauric acid trihydrate (HAuCl_4_.3H_2_O) was used as the Au+3 ion precursor for gold nanoparticles. Deionized water was used during the experiments. *Micromeria biflora* was collected from Swat Malakand Division, Thana, Betkhaila District, KPK, Pakistan. A specimen was kept in the herbarium of Botany Department of Govt. Degree College Hayatabad Peshawar with the voucher No. Bot.2020–3(MB) after identification by Professor Amanullah (taxonomist).

### 4.2. Preparation of Crude Flavonoids Micromeria Extract

The plant was dried in the shade, crushed, and impregnated with n-hexane for a day before being extracted with n-hexane by means of a Soxhlet extractor to remove all terpenes in order to make the extract non-sticky. After degreasing, the plant was soaked (methanol/water 30:70) under ambient conditions for two days before extracting the flavonoids with a Soxhlet apparatus. The flavonoids extract was then concentrated at 50 °C in rotary evaporator untill all the methanol was removed. The extract was preserved in a 100 mL flask as a stock solution having flavonoids content of 120 mg/mL for future use.

### 4.3. Preparation of Amoxicillin Solution

The amoxicillin antibiotic was purchased from Glaxo Smith Pharma (London, UK) and used in the synthesis of AuNPs amoxicillin conjugates (AuNP-amoxi). One mili molar (1 mM) solution of amoxicillin was prepared by dissolving 0.41 mg of powdered amoxicillin in 100 mL of double deionized water and kept in 100 mL volumetric flask for further experimentation.

### 4.4. Synthesis of AuNPs Using Crude Flavonoids Extract of Micromeria biflora

The synthesis of AuNPs was accomplished by taking varying amounts of tetrachloroaurate trihydrate against a fixed volume of flavonoids extract in 100 mL Erlenmeyer flask and stirring the solution for 3 h at room temperature. During stirring, the color of the solution changed from yellow to green and then to ruby red, indicating the formation of AuNPs. This was also supported by UV–visible spectroscopy.

#### Synthesis of Gold Nanoparticles-Amoxicillin Conjugate (AuNPs-amoxi)

A well-known protocol with slight changes was adopted [30]. In a 100 mL Erlenmeyer flask, 20 mL of AuNPs solution was mixed with 1 mL of 1 mM amoxicillin solution, and the mixture was stirred for 72 h at room temperature. To remove free drug molecules, the sample was centrifuged at 18,000 rpm, and the residue was suspended in distilled water. UV–visible spectroscopy confirms the formation of AuNPs-amoxicillin conjugates (Au-amoxi).

### 4.5. Characterization Techniques

The initial synthesis of AuNPs and Au-amoxi conjugate was confirmed by analyzing SPR (surface plasmon resonance) peaks using UV–vis spectrophotometry (Hitachi U-3200, Tokyo, Japan). The surface morphologies were confirmed with SEM (JSM 5910 JEOL, Tokyo, Japan). The elemental composition of AuNPs and AuNPs-amoxi was confirmed by EDX (INC-200, Oxford Instruments, Abingdon, UK), while the crystalline structure was confirmed with XRD (JDX-9C-XRD, Tokyo, Japan) and FT-IR (Prestige 21 Shimadzu, Kyoto, Japan).

### 4.6. Bioactivities Tests

#### 4.6.1. In Vivo Studies: Anti-Inflammatory and Antinociceptive Activities

Animal: The Department of Pharmacy, University of Peshawar, provided healthy BALB/c mice of either sex (weight 25–30 g), which were retained in metal cages for seven days (22 ± 2 °C with a 12 h light/dark cycle). They had unlimited access to food and drink. The study protocol was approved by the institutional Ethical Committee under application number 09/EC/F.LIFE-2020.

##### In Vivo Carrageenan-Induced Hind Paw Edema Model

The anti-inflammatory activity was performed according to reported protocol [52,53]. The animals were divided into six sets, and each set included six animals. SET I animals received a single dose of vehicle (3% DMSO, 1% Tween-80, and 96% physiological saline) combined with carrageenan as a negative control. SET II animals received diclofenac (50 mg/kg) as a positive control, while other SETs (III-VI) received doses of AuNPs and Au-amoxi conjugate (5 mg/kg and 10 mg/kg), amoxicillin (50 mg/kg and 100 mg/kg), and plant extract (P.E) (50 mg/kg and 100 mg/kg), respectively. Animals were injected with carrageenan (0.05 mL; 1%) solution via the subplantar area of the left hind paw, and after 60 min of treatment, the paw volume was measured using a digital plethysmometer (Plan lab, Spain) at different time intervals (1 h, 3 h, and 5 h). Percentage inhibition was measured using Equation (1).

(1) $$\%\;Inhibition=\frac{A-B}{A}\times 100$$

where A and B are the increase in paw volume of control and test treatment sets, respectively.

#### 4.6.2. Antinociceptive Activities

The antinociceptive activities of the synthesized AuNPs and its amoxi conjugates were evaluated using the following tests.

##### Acetic-Acid-Induced Writhing Test

Albino mice of both sexes weighing 18–22 g were used in this study. All animals were fasted for 2 h before testing. Animals were divided into six groups (n = 6). As a control, group I received an intraperitoneal injection of vehicle, while group II received standard diclofenac sodium (20 mg/kg body weight), and the other groups received AuNP and Au-amoxi (5 and 10 mg/kg, i.p), amoxicillin (50 and 100 mg/kg, i.p), and P.E (50 and 100 mg/kg, i.p). After 30 min, the animals were injected with 1% acetic acid intravenously. After 5 min of acetic acid administration, the writhing was counted. For 20 min, the number of abdominal contractions (writhes) was counted (Figure 3). The percent antinociceptive effect was calculated from the number of writhes as

(2) $$\%Inhibition=\frac{1-test}{Vehicle}\times 100$$

##### Hot Plate Test

This test is commonly used for the evaluation of the central antinociceptive ability of the drug and compounds (Brochet et al., 1986). The pain-induced effect of the test drug (Amoxicillin), AuNPs, Au-amoxi, and flavonoids extract was tested in mice using a hot plate analgesiometer maintained at 54.0 ± 0.10°. The animals were divided into 6 groups, including vehicle 10 mL/kg (Group I), a standard analgesic Tramadol 30 mg/kg (Group II), test compounds, AuNPs, Au-amoxi (5 and 10 mg/kg), amoxicillin, and plant extract (50 and 100 mg/kg). The vehicle, Tramadol, and test compounds were injected intraperitoneally. Post treatment cut of time was 30 s. The animal’s withdrawal responses on the hot plate were noted at intervals of 30 min, 60 min, and 90 min. The %-antinociceptive activity was calculated from the latencies as:

(3) $$\%\mathrm{Pr}otection=\frac{test-baseline}{cutoff-baseline}\times 100$$

Statistical Analysis: Data were analyzed by one-way ANOVA followed by Tukey’s test using Graph pad prism software. A *p*-Value of ≤0.001 was considered statistically significant.

## 5. Conclusions

We have developed green gold using flavonoids extract of *M. biflora* as reducing and stabilizing agent, and subsequently conjugated these AuNPs with amoxicillin without using any additional linkers. The AuNPs average size is 42–78 nm, while the Au-amoxi conjugate was 45–113 nm. The FT-IR analysis shows the possible involvement of HN_2_ or OH group for the formation of AuNPs while carbonyl groups are responsible for its stabilization. Good anti-inflammatory activities along with significant reduction in pain shown by both AuNPs and Au-amoxi reveal that they can be potentially used as drugs and prodrugs for biomedical applications. AuNPs and Au-amoxi conjugates may aid in the improvement of new therapeutic drugs against pathogens and the treatment of severe pathological conditions by reducing the side effects of synthetic medicine in the future.

## Data Availability

All the available data incorporated in the MS can be found with K.J. and S.A.

## Sample Availability

Samples of the compounds are available from the first author.
